# Heart Rate and Acceleration Dynamics during Swim-Fitness and Stress Challenge Tests in Yellowtail Kingfish (*Seriola lalandi*)

**DOI:** 10.3390/biology13030189

**Published:** 2024-03-15

**Authors:** Arjan P. Palstra, Wout Abbink, Wisdom E. K. Agbeti, Leo Kruijt, Pauline Jéhannet, Martin J. Lankheet

**Affiliations:** 1 Animal Breeding and Genomics, Wageningen University & Research, 6700 AH Wageningen, The Netherlands; wout.abbink@wur.nl (W.A.); wisdom.agbeti@wur.nl (W.E.K.A.); leo.kruijt@wur.nl (L.K.); pauline.jehannet@wur.nl (P.J.); 2 Experimental Zoology Group, Wageningen University & Research, 6700 AH Wageningen, The Netherlands; martin.lankheet@wur.nl

**Keywords:** swimming exercise physiology, aquaculture, amberjack, metabolic rate, sensor implants, precision farming

## Abstract

**Simple Summary:**

The yellowtail kingfish is a highly active and fast-growing marine fish with promising potential for aquaculture. In this study, we learned about the energy that these fish spend by measuring heart rate and acceleration during both a test where fish had to swim at increasing speeds and a test where fish were subjected to four steps of increasing stress load. Yellowtail kingfish need to swim to provide sufficient water flow over the gills to be able to extract sufficient oxygen. Oxygen consumption, heart rate and acceleration all increased when the fish had to swim faster. The oxygen levels that the fish consumed when swimming were high and heart rate values were even among the highest ever measured for fishes. Oxygen consumption and heart rate were only correlated when fish were swimming steadily near the optimal swimming speed, which is when all of their energy is required for fueling the muscles. With every stress step, the acceleration peak decreased, possibly suggesting that fish knew what was coming, until the fourth step, when fish were also chased, where the acceleration and heart rate showed their highest peaks. Acceleration and heart rate data can be used for modelling the energy flows that are required for steady swimming and unsteady stress movements. Such an energy economy model can then be applied for estimating the energy that the fish spend in field studies and for optimal farming of this species by exercising them.

**Abstract:**

The yellowtail kingfish is a highly active and fast-growing marine fish with promising potential for aquaculture. In this study, essential insights were gained into the energy economy of this species by heart rate and acceleration logging during a swim-fitness test and a subsequent stress challenge test. Oxygen consumption values of the 600–800 g fish, when swimming in the range of 0.2 up to 1 m·s^−1^, were high—between 550 and 800 mg·kg^−1^·h^−1^—and the heart rate values—up to 228 bpm—were even among the highest ever measured for fishes. When swimming at these increasing speeds, their heart rate increased from 126 up to 162 bpm, and acceleration increased from 11 up to 26 milli-g. When exposed to four sequential steps of increasing stress load, the decreasing peaks of acceleration (baseline values of 12 to peaks of 26, 19 and 15 milli-g) indicated anticipatory behavior, but the heart rate increases (110 up to 138–144 bpm) remained similar. During the fourth step, when fish were also chased, peaking values of 186 bpm and 44 milli-g were measured. Oxygen consumption and heart rate increased with swimming speed and was well reflected by increases in tail beat and head width frequencies. Only when swimming steadily near the optimal swimming speed were these parameters strongly correlated.

## 1. Introduction

The yellowtail kingfish, *Seriola lalandi* (Valenciennes, 1833), is a fast-swimming predatory fish that migrates over long distances [1]. It is a fast-growing athlete that is successfully farmed in sea pens, as well as in marine recirculating aquaculture systems [2,3], although the energy economy under these conditions of fast growth, high feed intake and high density poses metabolic challenges. Induced flow could contribute to successful performances in terms of growth, health and welfare [4,5]. Swim-training of yellowtail kingfish at optimal speed enhances body growth, lowers feed conversion ratio, increases cardiac output capacity [6] and, therefore, represents a promising approach for application at the farm.

The energy economy of fishes, compromising the whole range of active metabolism, can be established by subjecting fish to swim-fitness tests and stress challenge tests, encompassing the more steady and unsteady movements, respectively. A swim-fitness test generally involves swimming fish at increasing speeds in a swim tunnel (e.g., [6,7,8]). The duration of the test and the speed at each interval determine the aerobic and anaerobic component of the metabolic performance, reflecting the contributions of the red and white skeletal muscle [9], respectively. The aerobic component can be quantified by measuring the oxygen consumption. Locomotory behavior analysis can explain the observed variations in oxygen consumption. A stress challenge test generally involves subjecting the fish to a standardized stressor such as netting (e.g., [10]), crowding (e.g., [11]), lowering water level (e.g., [12]) or chasing (e.g., [13]), and measuring the cortisol response. Svendsen et al. [14] recently published an elegant, graded stress challenge test with four stress induction steps combining the lowering of the water level with chasing during the fourth step, and applying it on farmed Atlantic salmon, *Salmo salar* (Linnaeus, 1758).

Telemetry to monitor fish movement has a long research history of tracking the iconic fish migrations of eels (e.g., [15]), salmons (e.g., [16]), sturgeons (e.g., [17]), tunas (e.g., [18]) and sharks (e.g., [19]). More recent is the use of acoustic sensors for measuring real-time acceleration, used as a proxy for activity [20,21,22,23,24]. In addition to acceleration, loggers can measure heart rate, although the size of the logger with the combined sensor allows use only on larger fish (>400 g), and the loggers need to be retrieved for the purpose of data collection. On an experimental scale, sensors have been developed and applied to measure respiration [25], blood glucose [26,27], and to calculate blood oxygen saturation [28]. Collection of such physiological parameters allows mapping of the energy economy of free-swimming fish, which could help with filling biological knowledge gaps, determining the impacts of climate change and contributing to precision farming.

In this study, we attempted to gain more insight into the energy economy of yellowtail kingfish by investigating heart rate and acceleration, as determined by the application of Star-Oddi loggers at increasing swimming speeds during a swim-fitness test, and during an induced stress challenge test, which entailed the repeated lowering of water levels [14]. Oxygen consumption was measured, locomotory behavior (including tail beats and respiration frequencies) was monitored by high-speed camera recordings during the swim-fitness test, and parameters were related to heart rate and acceleration. Stress induction steps were validated by water cortisol measurements. Ultrasound was used as an additional determinator of heart rate, although under anesthesia. Herewith, we aimed to answer two research questions: (i) What are the heart rate and acceleration of yellowtail kingfish during graded swimming and stress loads? (ii) How do heart rate and acceleration relate to oxygen consumption and locomotory behavior during swimming at increasing speeds?

## 2. Materials and Methods

### 2.1. Ethical Approval

Experimental protocols complied with the current laws of The Netherlands and were approved by the Dutch Central Committee for Animal Experiments (CCD), project number AVD401002016652, 12 December 2016, and by the Animal Experiments Committee (DEC) and Authority for Animal Welfare (IvD) of Wageningen University and Research, experiment number 2016.D-0039.006, 1 October 2021.

### 2.2. Experimental Fish and Conditions

Yellowtail kingfish (N= 30, ~500 g) from the farm Kingfish Company (Kats, The Netherlands) were transported to the animal experimental facilities of CARUS in Wageningen (The Netherlands). The fish were housed in two circular 1200 L tanks, with a diameter of 158 cm, connected to a marine recirculating aquaculture system (RAS). The experimental conditions were the same as those the fish experienced at the farm: the salinity of the seawater was 34.3 ± 1.9 ppt, the temperature was kept stable at 23.9 ± 0.8 °C, and a 20 h light: 4 h dark regime was maintained. Fish were fed at a 12 g·kg^−0.8^ ration with commercial pellets (Biomar, Aarhus, Denmark; Inicio Plus, 6 mm) by automated feeding belts. After two weeks, N = 16 fish were randomly selected for surgical implantation of the loggers. The remaining N = 14 fish were considered as the controls without a logger. Groups were kept separate in two new, circular 700 L tanks (diameter 120 cm; marine RAS). The water quality over the whole experimental period remained well in the tanks with values of NH_4_^+^ ~0 mg·L^−1^, NO_2_^−^ 0.43 ± 0.63 mg·L^−1^ and NO_3_^−^ 203 ± 135 mg·L^−1^, as measured weekly with a SmartChem analyzer (Hamburg, Germany) and in-between with drop tests.

### 2.3. Heart Rate and Acceleration Loggers

**Figure 1:**
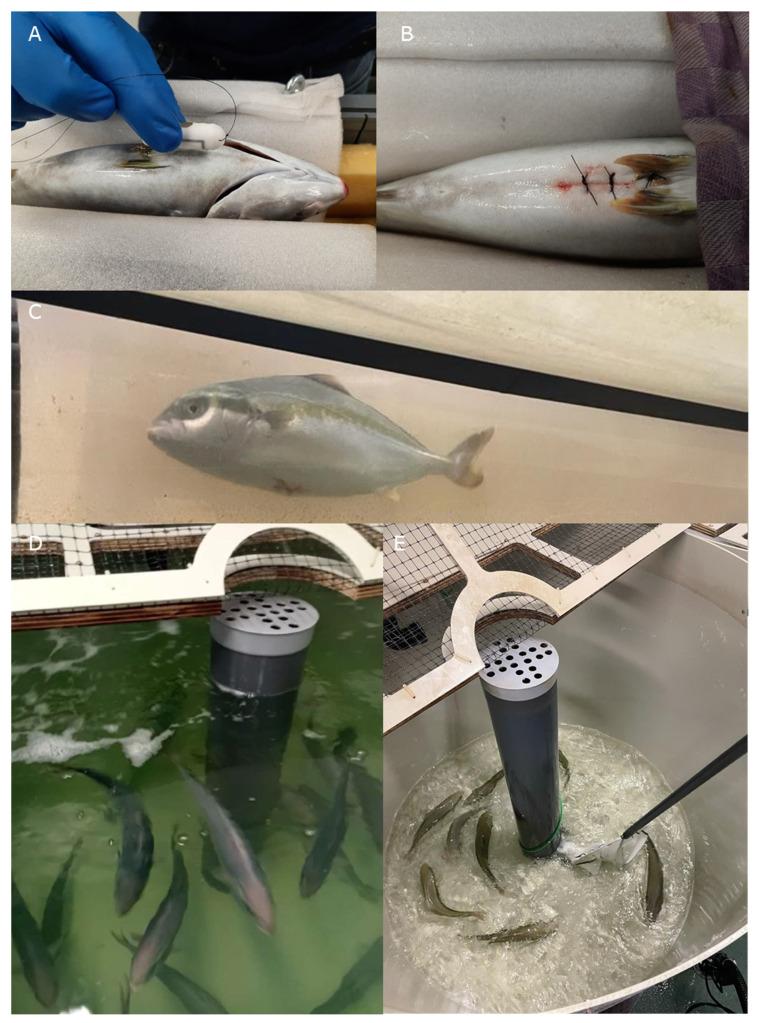
Overview of the experimental approach, showing (**A**) the position where the logger was implanted, (**B**) the stitch to anchor the logger and two additional stitches to close the wound, (**C**) a swimming yellowtail kingfish with implanted logger in the swim tunnel where, additional to heart rate and acceleration, oxygen consumption and locomotory behavior were also monitored, (**D**) the fish still at high water level and (**E**) at low water level, inducing stress.

DST milli-HRT ACT (Star-Oddi, Gardabaer, Iceland) loggers (for more information Ref. [29]) were implanted (Figure 1A,B) in fish at a size of 536 ± 92 g body weight (BW) and 34 ± 2 cm total length (TL). These loggers, hermetically sealed for biocompatibility, were 3.9 cm long and 1.3 cm wide and weighed 12 g. Fish were anesthetized with phenoxyethanol (0.3 mL·L^−1^) in aerated water. When anesthetized, the fish was placed on a surgical table with the ventral side up (Figure 1A,B). This was done under continuous flow of water with a low dose of phenoxyethanol (0.15 mL·L^−1^) passing over the gills. Prior to the incision, 100 μL of lidocaine was injected at the incision area for pain relief. An incision of about 1–2 cm was made with a scalpel on the ventral side at the edge of the pelvic fins, through which the disinfected logger was inserted in the abdominal cavity. The logger was positioned in the direction of the pericardial cavity and as close to it as possible, and then anchored with a twice-knotted single suture (Ethilon 3-0 669H, Ethicon, NJ, USA; FS-1 24 mm 3/8c reverse cutting needle and 75 cm black monofilament non-absorbable suture). The incision was then stitched with two single sutures and betadine was applied to prevent infection. Fish were sufficiently anesthetized after 8 ± 2 min, surgery lasted for 8 ± 2 min, and fish were swimming again after 2 ± 1 min. Implanted fish were allowed to recover for a minimum of 12 days in the 700 L tank that was connected to the same RAS system before the experiments were started.

Loggers were recovered and data downloaded after experimentation. Heart rate (HR) was derived by manual annotation from the electrocardiogram (ECG) signal at 200 Hz, recorded every 10 min for 7.5 s as beats per minute (bpm) using HRT-Analyzer v.1.0 software from Star-Oddi and a custom filter file for quality improvement. For each swim speed, six heart rate measurements were obtained, and all were used to set the heart rate for that particular swimming speed. The baseline heart rate was calculated by averaging 30 heart rate recordings throughout the days before the experiments. Acceleration (ACC) was measured as external acceleration (EA), which is the recorded three-axis acceleration above standard gravity defined with static calibration, normalized, and calculated as the vectorial sum in milli-g (more detailed description in [30]). Reported is the average external acceleration value (AvEA, which is an average of 600 3-axis measurements over 1 min), recorded at 10 Hz.

### 2.4. Swim-Fitness Test and Respirometry

Three 127 L Blazka-type swim tunnels (Ref. [31] for a detailed description; Figure 1C) were used for the swimming tests. Tunnels were connected to a 400 L tank filled with water, which was aerated to maintain high oxygen levels. Water from the tanks was recirculated through the tunnels using EHEIM pumps (2400 L·h^−1^; EHEIM GmbH & Co. KG, Deizisau, Germany). The water inlet was closed by a valve during oxygen measurements. A bypass with MINI-DO galvanic cell oxygen probes in a 4-channel respirometry system with OXY-REG oxygen analyzers (DAQ-PAC-G4; Loligo Systems Aps, Tjele, Denmark) allowed measuring of the total oxygen content of the water in percentage, which dropped in the period that the valves were closed due to oxygen consumption of the fish (ΔO_2_%).

Fish (controls and fish with loggers, in random order) were swum in series of three during a period of 17 days. Fish were individually transferred from the tank, lightly anesthetized, measured in length and weight, and introduced in the swim tunnels. Earlier experience shows that overnight acclimation in the swim tunnels works adversely for yellowtail kingfish [6]. Therefore, fish were kept at rest and were then swimming at incremental swimming speeds of 0.2, 0.4, 0.6, 0.8 and 1.0 m s^−1^, each for one hour of duration. During the last 10 min at each swimming speed, just before it was increased, tunnels were flushed for 10 min to re-establish high oxygen levels. Fish were allowed to acclimatize to a newly set swimming speed for 10 min before the valves were closed again and oxygen measurements commenced. Hence, oxygen measurements were done for 40 min per swimming speed. The experimenter was constantly present to observe swimming behavior. Background oxygen consumption (tunnels without fish) was measured at all flow speeds after swim-fitness tests and extracted from the values measured with fish present. The solid blocking effect was calculated [32] and ranged between 0% at a swimming speed of 0.2 m·s^−1^ up to 2% at a swimming speed of 1.0 m·s^−1^, and was therefore considered negligible.

From the decreasing oxygen contents when valves were closed, the oxygen consumption (MO_2_ in mg O_2_·kg^−1^·h^−1^) was calculated using the following formula:

(1) $${\mathrm{MO}}_{2}=\left(\frac{\Delta O_{2}\%\left(DOmax\times \frac{L}{100}\right)}{t\times BW}\right)$$

where DOmax is the maximum amount of oxygen dissolved in seawater (in mg O_2_·L^−1^ corresponding to salinity and temperature measurements), L is the volume of the swim tunnel (127 L), t is the time in hours and BW is the body weight in kg.

The first N = 8 implanted fish were immediately dissected after the swim-fitness test to obtain the logger data. These fish were anesthetized as described before, dissected, the loggers extracted, and the data downloaded. The following N = 8 implanted fish were kept alive after the last swim-fitness test for subsequent stress challenge tests that followed 5 days later.

### 2.5. Locomotory Behavior during the Swim-Fitness Test

Yellowtail kingfish locomotion was filmed with a Basler 2040–90 um NIR USB3 camera at a frame rate of 75 frames per second and a 12 ms exposure time. Pixels were binned 2 × 2 to improve sensitivity by a factor of 4. The camera was positioned one meter below the center of the tunnel. Final images were 1024 × 512 pixels at a resolution of 7.2 pixels per cm. A translucent back projection screen was placed on top of the tunnel to disperse the room lights into a homogeneous grey background. The camera view covered the full length and width of the tunnel, except for the most upstream 5 cm and downstream 10 cm. Custom software developed in Python, including the OpenCV image analysis library, was used to detect and save the fish contour in real-time. To detect the fish, we used a Gaussian blur (3 pix) filter to reduce noise, followed by a histogram normalization to improve the contrast in the images, and by a luminance threshold that selected dark objects on a light background. The fish were selected from detected objects (using the find_contours routine) based on the surface area and length-width ratio of the contour. We used a standard Kalman filter in OpenCV with position, speed and acceleration estimates to obtain smoothed estimates of fish tracks, quantified by the center of mass of the contour. Timing information, x and y locations and full body contours were saved to disk for offline analysis. The midline of each fish was analyzed from the saved contours based on a distance transform. For further details on detecting the snout of the fish and tracking the central axis, we refer to Arechavala-Lopez et al. [24]. The resulting axis was slightly smoothed using a univariate spline (k = 3, s = 5) for x and y data separately. To quantify tail beat parameters, we selected a point in the tail at about 0.8 × the standard length (SL) of the fish and we determined the lateral excursion relative to the midline through the head. Tail beat frequency (TBF) and amplitude (TBA) were obtained by performing a spectral analysis on the tail excursion as a function of time. Spectrograms were calculated with a temporal window size of 0.85 s (64 frames), shifted frame by frame. The resolution in the frequency domain was increased by padding the signal with zero values to a length 8 times that of the original signal. Frequency and amplitude were determined based on the maximum in the spectrogram at each frame. A similar calculation was performed on the width of the head, at the location of the opercula, to obtain frequency (HWF) and amplitude (HWA). These should reflect the rhythmic movements of the opercula, which can be different from swimming frequencies.

### 2.6. Stress Challenge Test and Cortisol

During the stress challenge, N = 8 implanted fish were kept with the controls in a tank and were then exposed to the following four conditions: reducing water level and (1) filling up immediately, (2) after 1 min, (3) after 5 min, and (4) after 5 min in combination with chasing the fish with a net (Figure 1D,E), with each iteration executed on the hour (at 9, 10, 11 and 12 h), according to the protocol published by Svendsen et al. (2021) [14]. At the end of each water reduction period, a 1 L water sample was taken in a 1 L bottle that was stored at −20 °C for later cortisol measurements.

After thawing, the samples were filtered, first through a fiberglass filter (SLAP02550; MilliporeSigma, Burlington, MA, USA) and then through a 0.45 μM Stericup Quick Release-HV Sterile Vacuum Filtration System (S2HVU11RE; MilliporeSigma, Burlington, MA, USA), and refrozen. After thawing, the exact net volume was measured by weighing. To concentrate the cortisol, the sample was flown through an Oasis HLB Plus LP extraction cartridge with 225 mg sorbent per cartridge (186000132; Waters, Milford, CT, USA), previously activated with 5 mL methanol and washed with 5 mL MilliQ water, using a vacuum manifold system. Cortisol was eluted with 3 mL ethanol. Ethanol was evaporated to dryness in a water bath (45 °C) under a stream of air. The residue was dissolved in 100 μL water and extracted with 2 mL diethyl ether by vortexing for 1 min, freezing the water phase, decanting diethyl ether phase into another tube, and allowing diethyl ether to evaporate to dryness in a water bath at 45 °C under a stream of air. This extraction was repeated 4 times. The final evaporated residue was dissolved in 200 μL assay buffer of the cortisol ELISA. Cortisol ELISA was performed according to the protocol of the Fish Cortisol ELISA Kit (CSB-E08487F_96; Cusabio, Houston, TX, USA) and samples were diluted 1/4 times, diluted in assay buffer.

### 2.7. Ultrasound

Nine days after the stress challenge test, the eight fish with loggers were individually anesthetized as described before and subjected to heart rate determination by ultrasound. An Esaote MyLabFive Vet ultrasonography unit (Esaote Europe BV, Maastricht, The Netherlands), with a 18 MHz LA435 ultrasound transducer and Pulsed Wave and color (CFM) Doppler, was used for this purpose. Afterwards, the fish were dissected, the loggers extracted, and the data downloaded.

### 2.8. Statistics

Data were analyzed using the R statistical package software, version 4.3.0. R packages lme4 [33] and lmerTest [34] were employed to fit models. Linear mixed models (LMM) were fitted to test the relations of MO_2_; TBA; TBF; HWA; HWF with swimming speed, with logger implantation as treatment and BW as covariate (formula: MO_2_~speed + treatment + BW). Fish ID was included as a random effect to account for individual variability. Similarly, LMM was fitted to test the relations of HR and ACC with swimming speed, also with BW as covariate and Fish ID as random effect. Non-significant effects of treatment and BW were removed from the model. Individual HR and ACC values during each stress induction step were analyzed by paired, one-tailed *t*-tests for significant increase from the baseline values, just preceding each stress induction step. Two-tailed Pearson correlation analyses were performed on all parameters at each swimming speed in SPSS 15. Data are presented as averages ± standard deviation.

## 3. Results

### 3.1. Swim-Fitness

When fish were subjected to the swim-fitness test, implanted fish measured 33 ± 2 cm SL and weighed 652 ± 152 g BW, and control fish measured 35 ± 2 cm SL and weighed 729 ± 122 g. The sizes of fish from both groups were not significantly different from each other, although there probably was impact of the surgery on growth during the recovery phase. Still, at the moment of implantation, fish measured 31 ± 2 cm SL and weighed 537 ± 97 g BW, showing significant growth between time of implantation and time of the swim-fitness test.

**Figure 2:**
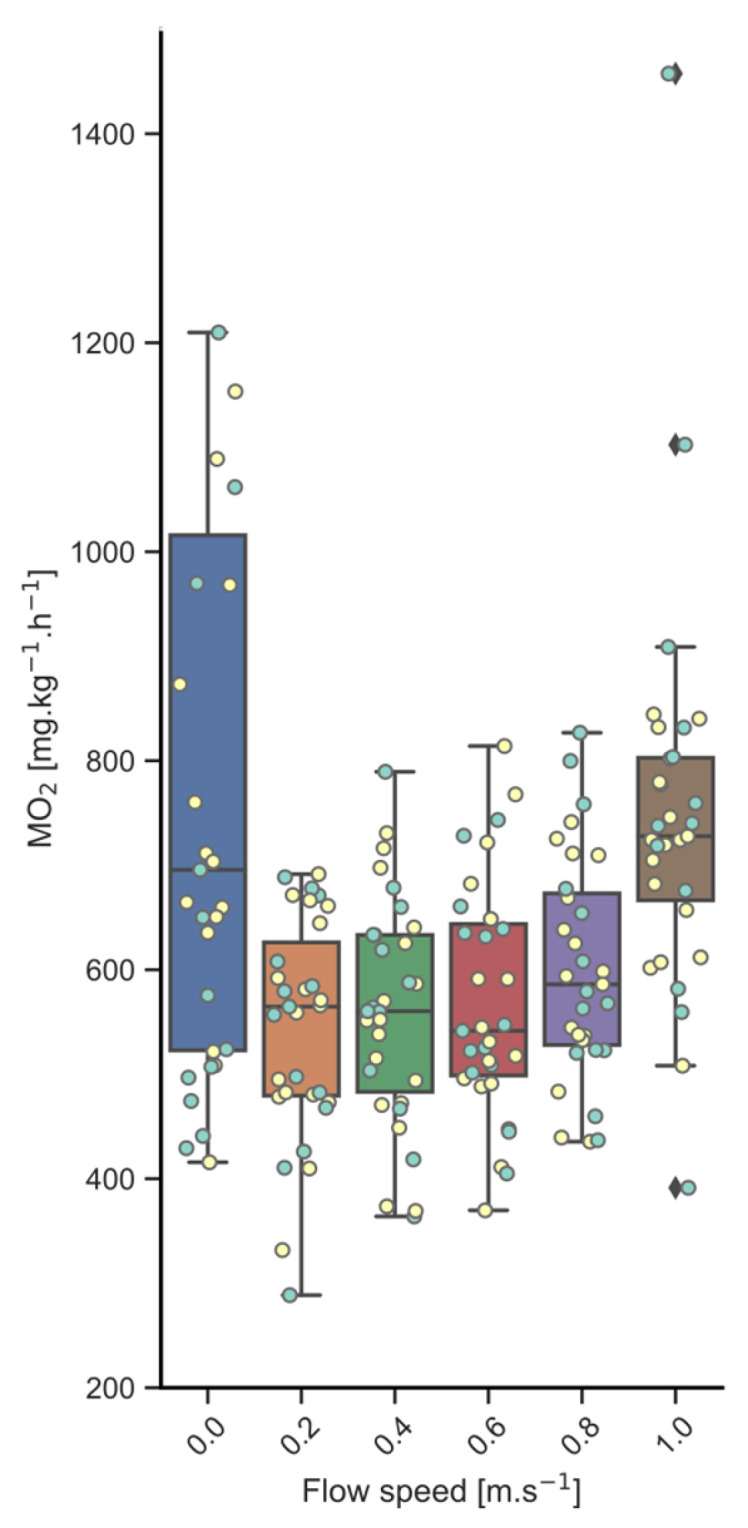
Oxygen consumption during the swim-fitness test. Shown are the MO_2_ values, in rest, with very high values for several fish, suggesting that oxygen consumption is problematic with restricted flow over the gills, and with swimming, where values are stable during the lower speeds of 0.2, 0.4 and 0.6 m·s^−1^, and then increase at higher speeds. No impact of the loggers was apparent between implanted fish (yellow datapoints) and the controls (blue datapoints). Bars include both implanted fish and controls.

Oxygen consumption (MO_2_; Figure 2) in rest was on average 675 ± 146 mg O_2_·kg^−1^·h^−1^ for implanted fish and 568 ± 171 mg O_2_·kg^−1^·h^−1^ for the controls, but for some fish, very high values were found, over 1 g O_2_·kg^−1^·h^−1^. When swimming, MO_2_ values were much lower again and more stable for all individual fish, with average values of:

- 550 ± 101 mg O_2_·kg^−1^·h^−1^ for implanted fish and 555 ± 93 mg O_2_·kg^−1^·h^−1^ for controls, when swimming at 0.2 m·s^−1^;
- 550 ± 109 mg O_2_·kg^−1^·h^−1^ for implanted fish and 573 ± 115 mg O_2_·kg^−1^·h^−1^ for controls, when swimming at 0.4 m·s^−1^;
- 566 ± 125 mg O_2_·kg^−1^·h^−1^ for implanted fish and 561 ± 92 mg O_2_·kg^−1^·h^−1^ for controls, when swimming at 0.6 m·s^−1^.
- At the higher swim speeds, MO_2_ levels increased up to:
- 595 ± 96 mg O_2_·kg^−1^·h^−1^ for implanted fish and 590 ± 108 mg O_2_·kg^−1^·h^−1^ for controls, when swimming at 0.8 m·s^−1^;
- 713 ± 94 mg O_2_·kg^−1^·h^−1^ for implanted fish and 786 ± 264 mg O_2_·kg^−1^·h^−1^ for controls, when swimming at 1.0 m·s^−1^.

All fish were kept swimming during the whole experimental period, and no fish fatigued. No significant differences existed between the implanted fish and their controls. Neither the variation in BW (*p* = 0.465) nor in implant treatment (*p* = 0.695) showed a significant influence on MO_2_. MO_2_ did not significantly increase with increasing speeds of 0.4 m·s^−1^ (*p* = 0.391) and 0.6 m·s^−1^ (*p* = 0.196). But, at speeds of 0.8 m·s^−1^ (*p* = 0.005) and 1.0 m·s^−1^ (*p* < 0.001), MO_2_ values were significantly higher.

The optimal swimming speed (Uopt) for implanted fish was 0.84 ± 0.06 m·s^−1^ at a cost of transport (CoT) of 169 ± 32 mg·kg^−1^·km^−1^, and for control fish Uopt was 0.82 ± 0.11 m·s^−1^ and CoT was 178 ± 40 mg·kg^−1^·km^−1^. There was no impact of the logger on Uopt and CoT between implanted fish and their controls.

### 3.2. Locomotory Behavior

**Figure 3:**
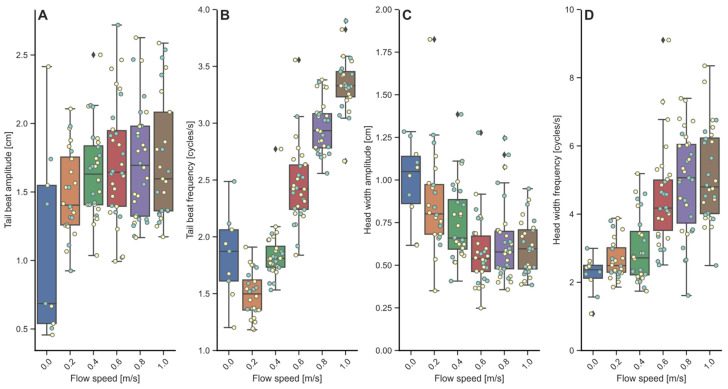
Locomotory behavior during the swim-fitness test. Shown are (**A**) Tail beat amplitude in arbitrary units (cm), which remains stable; (**B**) Tail beat frequency in cycles·s^−1^, which shows linear increase with speed; (**C**) Head width amplitude (cm), which is decreasing with speed and then stable at the higher speeds; and finally (**D**) Head width frequency, again in cycles·s^−1^, reflecting respiration, increasing with speed. No impact of the loggers on each of these parameters is apparent between implanted fish (yellow datapoints) and the controls (blue datapoints), except for head width frequency. Bars include both implanted fish and controls.

In general, tail beat amplitude remained fairly stable with increasing swimming speed (Figure 3A; 1.11 ± 0.70–1.74 ± 0.46 cm), but individual variations were high. TBA significantly increased with swimming speeds of 0.6 m·s^−1^ (*p* = 0.013), 0.8 m·s^−1^ (*p* = 0.021) and 1.0 m·s^−1^ (*p* = 0.004). During rest, values could become very low, down to 0.5 cm. The effect of implant treatment on TBA was not significantly different, but the effect of BW on TBA was significantly different and positive (*p* = 0.024).

Tail beat frequencies increased linearly with swimming speed from 1.49 cycle·s^−1^ at 0.2 m·s^−1^ to 3.35 cycle·s^−1^ at 1.0 m·s^−1^ (Figure 3B). The effects of BW and implant treatment were not significant. Swimming speed as a fixed effect accounted for about 87% of the variations observed in TBF, also exemplified by the strong positive effects of swimming speed on TBF (*p* < 0.001).

Head width amplitude decreased with swimming speeds in the lower range between 0 and 0.6 m·s^−1^ (0.93 down to 0.54 cm), and then remained stable at the highest swimming speeds of 0.8 and 1.0 m·s^−1^ (0.59–0.57 cm; Figure 3C). The effects of BW and implant treatment were not significant. Individual variations were very high and swimming speed accounted for just 19% of the variation.

Head width frequencies, expected to reflect respiration frequencies, and MO_2_ increased linearly with swimming speed, but were not significantly positively correlated (Figure 3D). HWF did increase significantly and positively, with swimming speeds of 0.6 up to 1.0 m·s^−1^ (*p* < 0.001). Implant treatment had a significantly positive effect on TWF (*p* = 0.024), while BW did not.

### 3.3. Heart Rate and Acceleration during Swim-Fitness and Stress Challenge Tests

**Figure 4:**
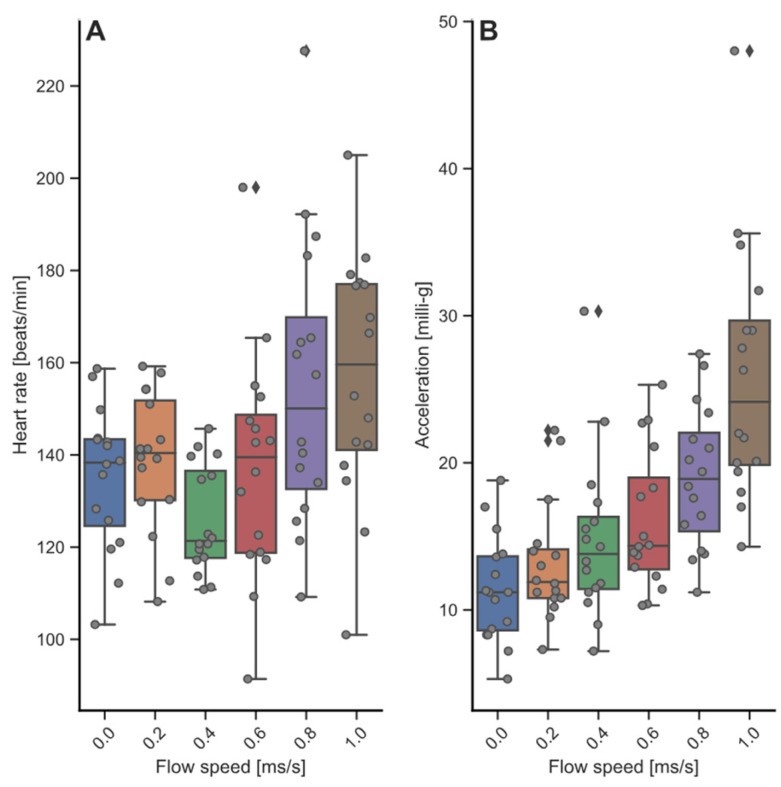
Heart rate and acceleration during the swim-fitness test. Shown are (**A**) Heart rate, with base line values of 110 bpm in the tank, between 126 to 139 bpm when swimming up to 0.6 m·s^−1^, and then increasing up to 155 bpm when swimming at 0.8 m·s^−1^ and 162 bpm at 1.0 m·s^−1^; (**B**) Acceleration, with base line values of 12 milli-g in the tank and linear increase, with swimming speeds up to 26 milli-g.

The base-line heart rate of fish in the tank was 110 ± 19 bpm and acceleration was AvEA = 12 ± 4 milli-g. The heart rate of fish in the swim tunnel was higher but stable between 126 to 139 bpm up to swimming at 0.6 m·s^−1^, and then increased up to 155 ± 2 bpm at 0.8 m·s^−1^ (*p* = 0.013) and 162 ± 7 bpm at 1.0 m·s^−1^ (*p* = 0.004; Figure 4A). Acceleration increased linearly with swimming speed from AvEA= 11 ± 1 to 26 ± 4 milli-g (Figure 4B). The increase was significant at speeds of 0.6 m·s^−1^ (*p* = 0.041), 0.8 m·s^−1^ (*p* < 0.001) and 1.0 m·s^−1^ (*p* < 0.001).

**Figure 5:**
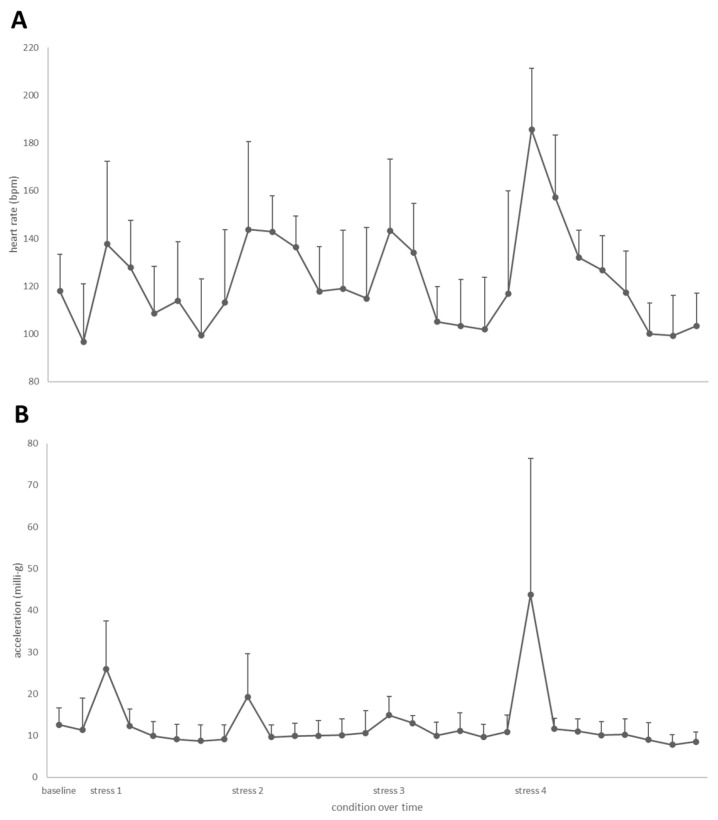
Heart rate and acceleration during the stress challenge test, with (**A**) heart rate values and (**B**) acceleration values. Shown on the *x*-axis are the baseline values, the in-between and the peak values at the subsequent stress induction steps 1 to 4, on the basis of Svendsen et al. [14].

The first, second and third lowering of the water level increased heart rates up to 138–144 bpm (Figure 5A), and acceleration was up to AvEA= 26 milli-g during the first stress induction but decreased to AvEA = 19 and 15 milli-g during the second and third stress induction (Figure 5B). The fourth lowering of the water level, which included the chasing, caused the highest heart rate at 186 bpm, with acceleration of AvEA = 44 milli-g. For all stress induction steps, heart rate and acceleration were increased, except for stress induction in step 3, where increases were just not significant (HR *p* = 0.08, ACC *p* = 0.07). When anaesthetized, the eight implanted fish that were subjected to the stress test had a heart rate of 47 ± 11 bpm, as determined by ultrasound.

### 3.4. Cortisol Concentrations during the Stepwise Stress Challenge Test

Cortisol concentrations in the system water increased more with each stress induction step from 197 pg·L^−1^ (baseline) to 245 pg·L^−1^ (step 1), then to 315 pg·L^−1^ (step 2), then to 411 pg·L^−1^ (step 3), and then to similar levels of 400 pg·L^−1^ after stress induction step 4, confirming the graded load nature of the stress challenge test.

### 3.5. Correlations between Parameters during the Swim-Fitness Test

Correlation analyses revealed swimming speed-dependent correlations between parameters reflecting size, respirometry, locomotory behavior and logger data (Table 1). Only the negative correlation between HWA and HWF was apparent for all swimming speeds. A positive correlation between MO_2_ and HR was only apparent when fish were swimming steadily at 0.6 and 0.8 m·s^−1^. Also, MO_2_ and HR were positively correlated to TBF when fish were swimming steadily at 0.6 m·s^−1^. Negative correlations between BW and HR, and between ACC and HWA, occur when fish were swimming at the highest speeds.

## 4. Discussion

In this study, we have collected unique heart rate and acceleration data of yellowtail kingfish during swimming at increasing speeds and under stress. While the fish were swimming, we also collected oxygen consumption and locomotory behavior data to relate to heart rate and acceleration data and obtain a comprehensive overview of kingfish energy economy.

An important condition was that the loggers (including the surgery procedure) would not have any significant impact on the measured parameters in our experimental fish. There were no significant differences in oxygen consumption and locomotion parameters, justifying the application of the loggers for this purpose. Optimal swimming speeds (Uopt implanted fish 0.84 m·s^−1^ vs. controls 0.82 m·s^−1^) and cost of transport (CoT implanted fish 169 mg·kg^−1^·km^−1^ vs. controls 178 mg·kg^−1^·km^−1^) were very similar.

An important observation was the very high oxygen consumption of several kingfish individuals under resting conditions. Seven fish (3 with logger, 4 without logger) had values higher than 1 g O_2_·kg^−1^·h^−1^, values higher than when swimming at 1 m·s^−1^, showing that they could not cope well with the individual housing in a swim tunnel with only flow-through and no additional flow created by the propellor. As soon as extra flow was generated and fish were swimming, oxygen consumption levels stabilized. This observation shows that acclimation in the swim tunnels under resting conditions does not work well for kingfish physiology. It also shows the importance of having sufficient dissolved oxygen and flow when farming this species. Kingfish requires sufficient water flow over the gills to meet oxygen demands; in other words, they need to swim. This also becomes apparent when observing fish tanks at the farm where fish are swimming continuously. Our finding stresses the importance of providing optimal exercise regimes for kingfish at the farm, making use of extra pumping capacity or propellors to create the required flows, or further developing methodology that can encourage them to swim faster, such as making use of the optomotor response [35].

During swimming in the range of 0.2 up to 1 m·s^−1^, the oxygen consumption values between 550 and 800 mg·kg^−1^·h^−1^ of the 600–800 g fish are well in line with those from our earlier study with smaller fish (844–1099 mg·kg^−1^·h^−1^ for 392 g fish; [6]), and with larger fish swimming at high speeds (656 and 799 mg·kg^−1^·h^−1^ for 2100 g fish at 20 and 25 °C, respectively, when swimming at 2.3 body lengths per second) from the study of Clark & Seymour [36]. These rates are in the upper range of other active fishes [37], but are still far below those of endothermic tuna species (1620–2700 mg·kg^−1^·h^−1^; [38,39]; also stated by [36]).

The heart rate values that we measured in this study are the highest for fish of this size. Maximal heart rate in fish as ectothermic ‘lower’ vertebrates generally do not exceed 100 to 120 bpm [40]. We measured maximal heart rates up to 228 bpm when swimming at 0.8 m·s^−1^, and 212 bpm at the fourth stress induction step. These values even exceed those of skipjack tuna (or bonito, *Katsuwonus pelamis*, Linnaeus, 1758), reported as the record holder of highest heart rate among adult fishes [40,41], with maximal heart rate up to 210 bpm [39,42]. They also exceed the earlier reported maxima of 135 bpm for 1 kg yellowtail kingfish under exhaustive stress by Morgenroth et al. [43]. Note that the method that is used to determine maximal heart rate should be taken into account, as more recent studies showed that free-swimming Atlantic salmon (~800 g) can have heart rate values up to 153 bpm [44] and Gilthead seabream (*Sparus aurata*, Linnaeus, 1758; ~500 g) can reach heart rate values as high as 172 bpm [45] during CTmax trials in tanks, which are higher than the previously reported values using either rapid screening protocol or combinations of respirometers and flow probes. Higher values have been reported for a much smaller fish, the tropical marine teleost *Bathygobius soporator* (Valenciennes, 1837; Gobbiidae), as determined by electrocardiography [46]. Still, in our study, fish swam up to speeds of 1 m·s^−1^, the maximum speed that could be reached with the used swim tunnels, which is below their critical swimming speed (Ucrit), as no fish fatigued. A follow-up study, using an experimental set-up and protocol to swim kingfish towards Ucrit, will give additional insights into maximum heart rate levels and swimming performance.

Baseline heart rate levels when showing spontaneous behavior, being housed group-wise in the tank, were 110 ± 19 bpm on average. When housed individually in the swim tunnel, heart rate was higher but stable between 126 to 139 bpm when swimming up to 0.6 m·s^−1^, and then increased up to 155 ± 2 bpm when swimming at 0.8 m·s^−1^, and 162 ± 7 bpm when swimming at 1.0 m·s^−1^. Clark & Seymour [36] also reported on a fairly stable and similar heart rate of ~80 bpm (at 20 °C) and ~120 bpm (at 25 °C) when swimming at speeds from 0.3 to 2.2 BL·s^−1^. Heart rate for larger yellowfin tuna (*Thunnus albacares*; Bonnaterre, 1788) was lower, between 30 and 130 bpm at 24 °C when swimming from 0.8 up to 2.9 FL·s^−1^ [47], and up to 109 bpm for bluefin tuna (*Thunnus thynnus*; (Linnaeus, 1758) when free-swimming in a sea cage [30].

As for stress loads, the first, second and third lowering of the water level led to stable peaks between 138 and 144 bpm, a 25% increase vs. baseline heart rate levels. The fourth lowering of the water level, which included the chasing, caused the highest heart rate at 186 bpm, a 70% increase vs. baseline heart rate levels. A similar relative increase of heart rate levels under stress was reported for Atlantic salmon where stress increased heart rates with 42–77% from 44–48 bpm up to 62–78 bpm [48].

Following our study on yellowtail kingfish, very similar swim and stress experiments were performed on Atlantic salmon and European seabass (*Dicentrarchus labrax*; Linnaeus, 1758) using the same experimental set-ups and loggers, allowing for future comparisons. Heart rate values of Atlantic salmon (31.8 ± 0.3 cm SL, 471 ± 11 g BW) were found to be much lower than for yellowtail kingfish and were very stable around 80–85 bpm when swimming in the range 0.2 up to 1.0 m·s^−1^ at 12 °C [49], similar to values of 79–86 bpm when peaking at the stress induction steps. Heart rate values of European seabass (28.3 ± 1.9 cm SL, 391 ± 61 g BW) were found to be in a similar range as Atlantic salmon of 64 up to 94 bpm on average, from baseline to swimming at a speed of 1.0 m·s^−1^ at 22 °C [50], and similar maximum values of 79–86 bpm when peaking at the stress induction steps.

When anaesthetized, the heart rate was down to 47 bpm, as determined by ultrasound. In general, heart rate reduction occurs under anesthesia (including by phenoxy ethanol, as in this study) due to the autonomous regulation of the heart [51], but this is not necessarily the case. Heart rate in rainbow trout (*Oncorhynchus mykiss*; Walbaum, 1792) is (initially?) higher under anesthesia than under regular conditions [52], an example of parasympathetic inhibition [53,54].

Acceleration increased with increasing swimming speeds, following a power function, just like for oxygen consumption. Interestingly, the predictability of a next stress induction step seemed to lower stress response, as reflected by acceleration, but not by heart rate. Acceleration went up to 26 milli-g during the first induction step, and was then lower at 19 and 15 milli-g during the second and third stress induction steps, respectively. Finally, with the fourth lowering of the water level that included the chasing, the highest acceleration was found at 44 milli-g. No studies have been published yet applying the Star-Oddi loggers in yellowtail kingfish, making direct comparison of acceleration difficult. Again referring to our swim and stress experiments on Atlantic salmon and European seabass using the same experimental set-ups and loggers for comparison, we found that salmon acceleration was in the range from 15.7 ± 1.2 to 26.3 ± 2.2 milli-g when swimming in the range 0.2 up to 1.0 m·s^−1^, and peaking to ~25 milli-g during the sequential stress induction steps [49]. Acceleration of European seabass was lower and ranged from 7.7 ± 4.6 to 21.2 ± 6.3 milli-g when swimming in the range 0.2 up to 1.0 m·s^−1^, and in the average range of 17 to 21 milli-g for the sequential stress induction steps [50].

Water cortisol measurements confirmed that the intended graded stress loads led to an increased cortisol stress response. With each stress induction step, the cortisol level increased, except for the fourth and last step, which was similar in peak level as the third step (400 pg·L^−1^). So, although the period of induced stress in a small water volume was much restricted in time (0, 1, 5, and 5 min), cortisol increases were clear for each of the stress induction steps. This shows that waterborne cortisol accumulates readily and can be accurately measured instantly (for review, see also [55]).

Oxygen consumption and heart rate increased linearly with swimming speed that was well reflected by tail beat frequency. Heart rate generally correlates strongly with O_2_ consumption rate [56,57]. From such strong positive correlations between oxygen consumption and heart rate, we can deduce the functional relation of extracting oxygen from the water to the blood and then pumping it by the heart throughout the body. With that, one parameter reflects the other two and relations can be used to calculate them. Still, positive correlations between MO_2_ and HR in this study were only apparent with swimming at speeds of 0.6 (slope 0.81, *p* < 0.001) and 0.8 m·s^−1^ (slope 0.61, *p* = 0.013), close to the optimal swimming speed of 0.85 m·s^−1^ for similarly sized fish, where energy investment in activities other than swimming has become minimal [6]. At lower and higher speeds, the line flattened and the correlation disappeared, coinciding with higher individual variations of both parameters. As Thorarensen et al. [58] showed by review, oxygen consumption can vary to a great degree while heart rate stays stable, as well as the other way around. While the contribution of heart rate to oxygen consumption during exercise may be limited, stroke volume and oxygen extraction efficiency could play a larger role. The ability to increase stroke volume is species dependent [59], causing corresponding variations in the increase in heart rate in response to exercise. The ability to increase stroke volume is also a plastic trait, as exercise training caused a significantly higher blood flow (31%) in the ventral aorta of yellowtail kingfish [6].

Particularly interesting is that the very high oxygen consumption under resting conditions was not accompanied by a high heart rate. Lack of flow over the gills may have triggered a hypoxic ventilatory response where fish tried to promote homeostasis by hyperventilating [60]. Fishes, contrary to tetrapods, generally do not display hypoxic tachycardia and even bradycardia [61], explaining the lack of heart rate increase. Increase in ventilation rate could not be confirmed by head width frequency, which was low and with relatively low variation. Head width frequency was expected to reflect the respiration frequency, but no correlations were found with oxygen consumption, nor with heart rate. Thus, both oxygen consumption and heart rate can provide important information about the physiological functioning of the fish. The relations between both need to be established in the lab before predictions of energy use on basis of measured heart rate are possible in aquaculture or in the field (also [62,63], respectively).

Importantly, heart rate and acceleration under swimming and stress conditions have now been mapped for kingfish, but the supervised analysis of the connection between acceleration patterns and spontaneous behaviors is still lacking. When mapped, acceleration readouts can be used to fingerprint certain behaviors, and can even be used as predictive biotic alarms for the farmer when conditions tend to go wrong. The first research studies in this direction on Seriola spp. have been published very recently and aimed to fingerprint spawning behavior [64] and several other behaviors [65,66].

## 5. Conclusions

The yellowtail kingfish is an athlete that needs to swim to provide sufficient water flow over the gills to meet its oxygen demands and has the highest heart rate among fishes ever reported to pump the oxygenated blood to the tissues. Only when swimming steadily nearing the optimal swimming speed are both parameters strongly correlated. Oxygen consumption and heart rate then increase with swimming speed, which is reflected by tail beat and head width frequencies. Heart rate and acceleration reflect exercise and stress loads very well. Predictability lowers stress, as reflected by activity but not by heart rate. Heart rate and acceleration are higher at maximum stress induction than at the maximum exercise induction in this study.

## Figures and Tables

**Table 1:** Swimming speed-dependent pairwise correlations. Indicated are the positive (green) and negative (orange) correlations and their significance level (light *p* < 0.05, dark *p* < 0.01). TL = total length (cm); SL = standard length (cm); BW = body weight (g); MO_2_ = oxygen consumption (mg·kg^−1^·h^−1^); HR = heart rate (bpm); ACC = acceleration (milli-g); TBA = tail beat amplitude (cm); TBF = tail beat frequency (cycles·s^−1^); HWA = head width amplitude (cm); HWF = head width frequency (cycles·s^−1^). MO_2_—HR correlations are apparent at speeds 0.6 and 0.8 m·s^−1^.

		Swimming Speed (m·s^−1^)					
		0	0.2	0.4	0.6	0.8	1
ACC	TBA	−0.960					
ACC	TBF	0.981					
SL	HR		0.615				
HWA	HWF		−0.582	−0.546	−0.679	−0.670	−0.550
TL	TBF			−0.382		−0.604	
SL	TBA			0.386		0.447	0.416
MO_2_	TBF				0.502		
HR	TBF				0.716		
MO_2_	HR				0.810	0.606	
TL	TBA					0.431	0.496
SL	TBF					−0.621	
BW	TBF					−0.543	
BW	TBA					0.367	0.437
BW	HR						−0.529
ACC	HWA						−0.662

## Data Availability

Raw data supporting the conclusions of this article will be made available by the authors, without undue reservation, to any qualified researcher.

## References

[1] Gillanders B.M., Ferrell D.J., Andrew N.L. (2001). Estimates of movement and life-history parameters of yellowtail kingfish (*Seriola lalandi*): How useful are data from a cooperative tagging programme?. Mar. Freshw. Res..

[2] Blanco Garcia A., Partridge G.J., Flik G., Roques J.A.C., Abbink W. (2015). Ambient salinity and osmoregulation, energy metabolism and growth in juvenile yellowtail kingfish (*Seriola lalandi* Valenciennes 1833) in a recirculating aquaculture system. Aquac. Res..

[3] Symonds J.E., Walker S.P., Pether S., Gublin Y., McQueen D., King A., Irvine G.W., Setiawan A.N., Forsythe J.A., Bruce m. (2014). Developing yellowtail kingfish (*Seriola lalandi*) and hāpuku (*Polyprion oxygeneios*) for New Zealand aquaculture. N. Z. J. Mar. Fresh..

[4] Palstra A.P., Planas J.V. (2011). Fish under exercise. Fish Physiol. Biochem..

[5] McKenzie D.J., Palstra A.P., Planas J., MacKenzie S., Bégout M.-L., Thorarensen H., Vandeputte M., Mes D., Rey S., De Boeck G. (2020). Aerobic swimming in intensive aquaculture: Applications for production, mitigation and selection. Rev. Aquacult..

[6] Palstra A.P., Mes D., Kusters K., Roques J.A.C., Flik G., Kloet K., Blonk R.J.W. (2015). Forced sustained swimming exercise at optimal speed enhances growth of yellowtail kingfish (*Seriola lalandi*). Front. Physiol..

[7] Palstra A., van Ginneken V., van den Thillart G. (2008). Cost of transport and optimal swimming speeds in farmed and wild European silver eels (*Anguilla anguilla*). Comp. Biochem. Physiol. A.

[8] Palstra A.P., Tudorache C., Rovira M., Brittijn B., Burgerhout E., van den Thillart G.E.E.J.M., Spaink H.P., Planas J.V. (2010). Establishing zebrafish (*Danio rerio*) as a novel exercise model: Swimming economy, swimming-enhanced growth and regulation of muscle growth marker gene expression. PLoS ONE.

[9] Videler J.J. (1993). Fish Swimming.

[10] Arends R.J., Mancera J.M., Munoz J.L., Bonga S.W., Flik G. (1999). The stress response of the gilthead sea bream (*Sparus aurata* L.) to air exposure and confinement. J. Endocrinol..

[11] Ganga R., Montero D., Gordon Bell J., Atalah E., Ganuza E., Vega-Orellana O., Tort L., Acerete L., Afonso J.M., Benitez-Sanatana T. (2011). Stress response in sea bream (*Sparus aurata*) held under crowded conditions and fed diets containing linseed and/or soybean oil. Aquaculture.

[12] Einarsdóttir I.E., Nilssen K.J. (1996). Stress responses of Atlantic salmon (*Salmo salar* L.) elicited by water level reduction in rearing tanks. Fish Physiol. Biochem..

[13] Madaro A., Olsen R.E., Kristiansen T.S., Ebbesson L.O., Flik G., Gorissen M. (2016). A comparative study of the response to repeated chasing stress in Atlantic salmon (*Salmo salar* L.) parr and post-smolts. Comp. Biochem. Physiol. A.

[14] Svendsen E., Føre M., Økland F., Gräns A., Hedger R.D., Alfredsen J.A., Uglem I., Rosten C.M., Frank K., Erikson U. (2021). Heart rate and swimming activity as stress indicators for Atlantic salmon (*Salmo salar*). Aquaculture.

[15] Wright R.M., Piper A.T., Aarestrup K., Azevedo J.M., Cowan G., Don A., Gollock M., Rodriguez Ramallo S., Velterop R., Walker A. (2022). First direct evidence of adult European eels migrating to their breeding place in the Sargasso Sea. Sci. Rep..

[16] Birnie-Gauvin K., Thorstad E.B., Aarestrup K. (2019). Overlooked aspects of the *Salmo salar* and *Salmo trutta* lifecycles. Rev. Fish Biol. Fisher..

[17] Brevé N.W., Vis H., Houben B., Breukelaar A., Acolas M.L. (2019). Outmigration pathways of stocked juvenile European sturgeon (*Acipenser sturio* L., 1758) in the Lower Rhine River, as revealed by telemetry. J. Appl. Ichthyol..

[18] Block B.A., Whitlock R., Schallert R.J., Wilson S., Stokesbury M.J., Castleton M., Boustany A. (2019). Estimating natural mortality of Atlantic bluefin tuna using acoustic telemetry. Sci. Rep..

[19] Araujo G., Agustines A., Tracey B., Snow S., Labaja J., Ponzo A. (2019). Photo-ID and telemetry highlight a global whale shark hotspot in Palawan, Philippines. Sci. Rep..

[20] Kolarevic J., Aas-Hansen Ø., Espmark Å., Baeverfjord G., Terjesen B.F., Damsgård B. (2016). The use of acoustic acceleration transmitter tags for monitoring of Atlantic salmon swimming activity in recirculating aquaculture systems (RAS). Aquacult. Eng..

[21] Føre M., Frank K., Norton T., Svendsen E., Alfredsen J.A., Dempster T., Eguiraun H., Watson W., Stahl A., Sunde L.M. (2018). Precision fish farming: A new framework to improve production in aquaculture. Biosyst. Eng..

[22] Muñoz L., Aspillaga E., Palmer M., Saraiva J.L., Arechavala-Lopez P. (2020). Acoustic telemetry: A tool to monitor fish swimming behavior in sea-cage aquaculture. Front. Mar. Sci..

[23] Palstra A.P., Arechavala-Lopez P., Xue Y., Roque A. (2021). Accelerometry of seabream in a sea-cage: Is acceleration a good proxy for activity?. Front. Mar. Sci..

[24] Arechavala-Lopez P., Lankheet M., Diaz-Gil C., Abbink W., Palstra A.P. (2021). Swimming activity of gilthead seabream (*Sparus aurata*) in swim-tunnels: Acoustic accelerometry, oxygen consumption and body motion. Front. Anim. Sci..

[25] Martos-Sitcha J.A., Sosa J., Ramos-Valido D., Bravo F.J., Carmona-Duarte C., Gomes H.L., Calduch-Giner J.À., Cabruja E., Vega A., Ferrer M.Á. (2019). Ultra-low power sensor devices for monitoring physical activity and respiratory frequency in farmed fish. Front. Physiol..

[26] Endo H., Yonemori Y., Musiya K., Maita M., Shibuya T., Ren H., Hayashi T., Mitsubayashi K. (2006). A needle-type optical enzyme sensor system for determining glucose levels in fish blood. Anal. Chim. Acta.

[27] Wu H., Shinoda R., Murata M., Matsumoto H., Ohnuki H., Endo H. (2019). Real-time fish stress visualization came true: A novel multi-stage color-switching wireless biosensor system. Biosens. Bioelectron..

[28] Svendsen E., Føre M., Randeberg L.L., Olsen R.E., Finstad B., Remen M., Bloecher N., Alfredsen J.A. (2023). ECG augmented pulse oximetry in Atlantic salmon (*Salmo salar*)—A pilot study. Comput. Electron. Agr..

[29] Bjarnason Á., Gunnarsson A., Árnason T., Oddgeirsson M., Sigmarsson A.B., Gunnarsson Á. (2019). Validation of ECG-derived heart rate recordings in Atlantic cod (*Gadus morhua* L.) with an implantable data logging system. Anim. Biotelemetry.

[30] Rouyer T., Bonhommeau S., Bernard S., Kerzerho V., Derridj O., Bjarnason Á., Allal H., Steffensen J.F., Deguara S., Wendling B. (2023). A novel protocol for rapid deployment of heart rate data storage tags in Atlantic bluefin tuna *Thunnus thynnus* reveals cardiac responses to temperature and feeding. J. Fish Biol..

[31] Van den Thillart G.E.E.J.M., Van Ginneken V., Körner F., Heijmans R., Van der Linden R., Gluvers A. (2004). Endurance swimming of European eel. J. Fish Biol..

[32] Bell W.H., Terhune L.D.B. (1970). Water tunnel design for fisheries research. Fish. Res. Board. Can. Tech. Rep..

[33] Bates D., Mächler M., Bolker B.M., Walker S.C. (2015). Fitting linear mixed-effects models using lme4. J. Stat. Softw..

[34] Kuznetsova A., Brockhoff P.B., Christensen R.H.B. (2017). lmerTest Package: Tests in Linear Mixed Effects Models. J. Stat. Softw..

[35] Herbert N.A., Kadri S., Huntingford F.A. (2013). A moving light stimulus elicits a sustained swimming response in farmed Atlantic salmon, *Salmo salar* L. Fish Physiol. Biochem..

[36] Clark T.D., Seymour R.S. (2006). Cardiorespiratory physiology and swimming energetics of a high-energy-demand teleost, the yellowtail kingfish (*Seriola lalandi*). J. Exp. Biol..

[37] Brett J.R. (1972). The metabolic demand for oxygen in fish, particularly salmonids, and a comparison with other vertebrates. Resp. Physiol..

[38] Brill R.W., Bushnell P.G. (1991). Metabolic and cardiac scope of high energy demand teleosts-the tunas. Can. J. Zool..

[39] Brill R.W., Bushnell P.G. (2001). The cardiovascular system of tunas. Fish Physiol..

[40] Lillywhite H.B., Zippel K.C., Farrell A.P. (1999). Resting and maximal heart rates in ectothermic vertebrates. Comp. Biochem. Physiol. A.

[41] Farrell A.P. (1996). Features heightening cardiovascular performance in fishes, with special reference to tunas. Comp. Biochem. Physiol. A.

[42] Bushnell P.G., Brill R.W. (1992). Oxygen transport and cardiovascular responses in skipjack tuna (*Kutsuwonas pelamis*) and yellowfin tuna (*Thunnus albacores*) exposed to acute hypoxia. J. Comp. Physiol..

[43] Morgenroth D., McArley T., Danielo Q., Harford A., Hickey A.J., Khan J., Sandblom E. (2022). Kingfish (*Seriola lalandi*) adjust to low salinity with only subtle effects to cardiorespiratory and growth performance. Aquaculture.

[44] Sandrelli R.M., Gamperl A.K. (2023). The upper temperature and hypoxia limits of Atlantic salmon (*Salmo salar*) depend greatly on the method utilized. J. Exp. Biol..

[45] Mignucci A., Bourjea J., Forget F., Allal H., Dutto G., Gasset E., McKenzie D.J. (2021). Cardiac and behavioural responses to hypoxia and warming in free-swimming gilthead seabream, *Sparus aurata*. J. Exp. Biol..

[46] Rantin F.T., Gesser H., Kalinin A.L., Guerra C.D.R., De Freitas J.C., Driedzic W.R. (1998). Heart performance, Ca^2+^ regulation and energy metabolism at high temperatures in *Bathygobius soporator*, a tropical marine teleost. J. Therm. Biol..

[47] Korsmeyer K.E., Lai N.C., Shadwick R.E., Graham J.B. (1997). Heart rate and stroke volume contributions to cardiac output in swimming yellowfin tuna: Response to exercise and temperature. J. Exp. Biol..

[48] Yousaf M.N., Røn Ø., Plebaniak Hagen P., McGurk C. (2022). Monitoring fish welfare using heart rate bio-loggers in farmed Atlantic salmon (*Salmo salar* L.): An insight into the surgical recovery. Aquaculture.

[49] Agbeti W.E.K., Palstra A.P. (2024). Heart Rate and Acceleration Logging during Swim-Fitness and Stress Challenge Tests in Atlantic Salmon Salmo salar.

[50] Tomàs J., Palstra A.P. (2024). Heart Rate and Acceleration Logging during Swim-Fitness and Stress Challenge Tests in European Seabass Dicentrarchus labrax.

[51] Brønstad A. (2022). Good Anesthesia Practice for Fish and Other Aquatics. Biology.

[52] Cotter P.A., Rodnick K.J. (2006). Differential effects of anesthetics on electrical properties of the rainbow trout (*Oncorhynchus mykiss*) heart. Comp. Biochem. Physiol. A.

[53] Randall D.J. (1962). Effect of an anaesthetic on the heart and respiration of teleost fish. Nature.

[54] Hill J.V., Davison W., Forster M.E. (2002). The effects of fish anaesthetics (MS222, metomidate and AQUI-S) on heart ventricle, the cardiac vagus and branchial vessels from Chinook salmon (*Oncorhynchus tshawytscha*). Fish Physiol. Biochem..

[55] Sadoul B., Geffroy B. (2019). Measuring cortisol, the major stress hormone in fishes. J. Fish Biol..

[56] Clark T.D., Sandblom E., Hinch S.G., Patterson D.A., Frappell P.B., Farrell A.P. (2010). Simultaneous biologging of heart rate and acceleration, and their relationships with energy expenditure in free-swimming sockeye salmon (*Oncorhynchus nerka*). J. Comp. Physiol. B.

[57] Lucas M.C. (1994). Heart rate as an indicator of metabolic rate and activity in adult Atlantic salmon, *Salmo salar*. J. Fish Biol..

[58] Thorarensen H., Gallaugher P.E., Farrell A.P. (1996). The limitations of heart rate as a predictor of metabolic rate in fish. J. Fish Biol..

[59] Farrell A.P., Eliason E.J., Sandblom E., Clark T.D. (2009). Fish cardiorespiratory physiology in an era of climate change. Can. J. Zool..

[60] Perry S., Jonz M., Gilmour K. (2009). Oxygen sensing and the hypoxic ventilatory response. Fish Physiol..

[61] Joyce W., Wang T. (2022). Regulation of heart rate in vertebrates during hypoxia: A comparative overview. Acta Physiol..

[62] Morgenroth D., Kvaestad B., Økland F., Finstad B., Olsen R.-E., Svendsen E., Rosten C., Axelsson M., Bloecher N., Føre M. (2024). Under the sea: How can we use heart rate and accelerometers to remotely assess fish welfare in salmon aquaculture?. Aquaculture.

[63] Cooke S.J., Brownscombe J.W., Raby G.D., Broell F., Hinch S.G., Clark T.D., Semmens J.M. (2016). Remote bioenergetics measurements in wild fish: Opportunities and challenges. Comp. Biochem. Physiol. A.

[64] Sakaji H., Hamada K., Naito Y. (2018). Identifying spawning events of greater amberjack using accelerometers. Mar. Biol. Res..

[65] Clarke T.M., Whitmarsh S.K., Hounslow J.L., Gleiss A.C., Payne N.L., Huveneers C. (2021). Using tri-axial accelerometer loggers to identify spawning behaviours of large pelagic fish. Movement Ecol..

[66] Clarke T.M., Whitmarsh S.K., Jaine F.R., Taylor M.D., Brodie S., Payne N.L., Butcher P.A., Broadhurst M.K., Davey J., Huveneers C. (2023). Environmental drivers of yellowtail kingfish, *Seriola lalandi*, activity inferred through a continental acoustic tracking network. Aquatic Conserv. Mar. Freshw. Ecosyst..

